# The importance of variant reinterpretation in inherited cardiovascular diseases: Establishing the optimal timeframe

**DOI:** 10.1371/journal.pone.0297914

**Published:** 2024-05-01

**Authors:** Anna Fernandez-Falgueras, Monica Coll, Anna Iglesias, Coloma Tiron, Oscar Campuzano, Ramon Brugada

**Affiliations:** 1 Department of Cardiology, Hospital Trueta, Girona, Spain; 2 Molecular Diagnostics and Personalized Medicine Unit, Clinical Laboratory, Hospital Trueta, Girona, Spain; 3 Cardiovascular Genetics Center, University of Girona-IDIBGI, Girona, Spain; 4 Centro de Investigación Biomédica en Red, Enfermedades Cardiovasculares (CIBERCV), Madrid, Spain; 5 Medical Science Department, School of Medicine, University of Girona, Girona, Spain; Shaheed Rajaei Hospital: Rajaie Cardiovascular Medical and Research Center, ISLAMIC REPUBLIC OF IRAN

## Abstract

Inherited cardiovascular diseases are rare diseases that are difficult to diagnose by non-expert professionals. Genetic analyses play a key role in the diagnosis of these diseases, in which the identification of a pathogenic genetic variant is often a diagnostic criterion. Therefore, genetic variant classification and routine reinterpretation as data become available represent one of the main challenges associated with genetic analyses. Using the genetic variants identified in an inherited cardiovascular diseases unit during a 10-year period, the objectives of this study were: 1) to evaluate the impact of genetic variant reinterpretation, 2) to compare the reclassification rates between different cohorts of cardiac channelopathies and cardiomyopathies, and 3) to establish the most appropriate periodicity for genetic variant reinterpretation. All the evaluated cohorts (full cohort of inherited cardiovascular diseases, cardiomyopathies, cardiac channelopathies, hypertrophic cardiomyopathy, dilated cardiomyopathy, arrhythmogenic cardiomyopathy, Brugada syndrome, long QT syndrome and catecholaminergic polymorphic ventricular tachycardia) showed reclassification rates above 25%, showing even higher reclassification rates when there is definitive evidence of the association between the gene and the disease in the cardiac channelopathies. Evaluation of genetic variant reclassification rates based on the year of the initial classification showed that the most appropriate frequency for the reinterpretation would be 2 years, with the possibility of a more frequent reinterpretation if deemed convenient. To keep genetic variant classifications up to date, genetic counsellors play a critical role in the reinterpretation process, providing clinical evidence that genetic diagnostic laboratories often do not have at their disposal and communicating changes in classification and the potential implications of these reclassifications to patients and relatives.

## Introduction

Inherited cardiovascular diseases comprise an extensive spectrum of genetic entities, including cardiomyopathies, principally hypertrophic cardiomyopathy (HCM), dilated cardiomyopathy (DCM) or arrhythmogenic cardiomyopathy (ACM); cardiac channelopathies mainly Brugada syndrome (BrS), long QT syndrome (LQTS), short QT syndrome (SQTS) and catecholaminergic polymorphic ventricular tachycardia (CPVT); and some valvular and aortic disorders such as Marfan syndrome. Incomplete penetrance and variable expressivity are hallmarks of inherited cardiovascular diseases leading to a heterogeneous clinical course. Early identification is crucial in prevention of malignant arrhythmias and sudden cardiac death (SCD), which may be the first manifestation of the disease [1–3].

Genetic testing is highly recommended in inherited cardiovascular diseases and SCD-associated cases as it is of great utility to confirm the diagnosis [2]. The detection of a pathogenic or likely pathogenic variant accounts as a diagnostic criterion, especially in channelopathies, thus the misclassification of a genetic variant can lead to an inappropriate genetic diagnosis and, consequently, to the adoption of unnecessary or inadequate therapeutic measures. For this reason, one of the main challenges when identifying a genetic variant continues to be its accurate classification.

Several publications have been available in these last two decades describing different methodologies and nomenclatures for genetic variant classification [4–8]. However, the wide range of variability to classification stressed the need to use a homogeneous system. Thus, different approaches have been put in place to determine which criteria and evidence are needed to be considered in order to reduce subjectivity in genetic variant interpretation. In 2015, the American College of Medical Genetics and Genomics (ACMG) and the Association for Molecular Pathology (AMP) published a joint consensus recommendation for the interpretation of genetic variants [9], which are currently extensively adopted. Nevertheless, classification discrepancies have been described between different laboratories and specialized professionals, even using these ACMG recommendations [10], highlighting the need to develop specifications for the appropriate use of each piece of evidence as well as specific gene and disease recommendations. Some of these specifications have already been published [11–16], leading to changes in the use of evidence and the strength with which they are applied and highlighting the variable nature of genetic variant classifications.

Reinterpretation of genetic variants refers to the process of reevaluating the interpretation or classification of a specific genetic variant in the context of new or updated evidence. Genetic variant reinterpretation could result in a reclassification, meaning the classification after reinterpretation differs from the originally reported classification. Currently, no timeframe is established for reinterpretation. Previous studies performed by our group suggested that variants classified not using the ACMG recommendations should be immediately reinterpreted [17] and suggested a maximum period of 5 years for the reinterpretation of variants classified according to the ACMG guidelines [18], while other authors have suggested lower time periods [19]. The potential benefit for the patients and their family members highlights the importance of reaching a consensus.

In this study, genetic variants reported during ten years in a cohort of inherited cardiovascular diseases have been reinterpreted to determine the frequency in which genetic variants should be re-evaluated and the impact it has on different inherited cardiovascular diseases subcohorts.

## Materials and methods

Genetic analyses performed in patients evaluated for cardiomyopathies, cardiac channelopathies or SCD at the Inherited Cardiovascular Unit of Hospital Trueta (Girona, Spain) from 1^st^ January 2012 to 31^st^ December 2021 were evaluated for genetic variant reinterpretation. During the last month of the inclusion period (December 2021), data from the genetic analyses were accessed to retrieve the reported genetic variants for the study purposes. An overall of 1425 genetic variants were identified in 618 patients. Genetic analysis was approved by the ethics committee of Hospital Trueta (Girona, Spain) following the World Medical Association Declaration of Helsinki. Written informed consent was obtained before genetic analysis. In minors, informed consent was obtained from parents or guardians.

Variants were categorized based on the patient’s phenotype into 3 groups: inherited cardiovascular diseases (full cohort 1425 variants), cardiomyopathies and cardiac channelopathies. Furthermore, both the cardiomyopathies and the cardiac channelopathies groups were each subdivided in 3 subcohorts: HCM, DCM and ACM, and BrS, LQTS and CPVT, respectively. The total reclassification rate was evaluated for all groups. In the full cohort and the cardiomyopathies and cardiac channelopathies groups, reclassification rates were also evaluated considering the year of the original report. Finally, in the different subcohorts, reclassification rates were evaluated considering the clinical validity of the genes.

Variants initially reported before the publication of the ACMG guidelines were originally classified following internal guidelines. When different nomenclature was used to name the classifications, an equivalence to the ACMG guidelines classifications was applied: pathogenic (P), likely pathogenic (LP), variants of uncertain significance (VUS), likely benign (LB) and benign (B) [9]. Variants initially classified as benign (B) and likely benign (LB) were not reinterpreted in this study due to their high global population frequency.

Genetic analyses were performed between 1^st^ January 2012 to 31^st^ December 2021 using three custom resequencing panels which interrogate genes related to cardiomyopathies, cardiac channelopathies or both for SCD cases, respectively. The genes included in each of the custom panels have been updated during the period of this study.

All 1425 genetic variants were reinterpreted at the end of the inclusion period (beginning in January 2022) following the ACMG recommendations and applying the updates that have been published since its publication. For most criteria, strength levels have been added to the initially described in the ACMG recommendations to be more specific with the essentially available evidence [12, 13, 20]. General population frequencies were obtained from the Genome Aggregation Database (gnomAD, https://gnomad.broadinstitute.org) and contrasted with the calculated BA1, BS1, and PM2 thresholds (criteria about variant allelic frequency) conforming to the corresponding penetrance, prevalence, and allelic or genetic contribution for the gene or disease [15, 21–23]. The PM2 criterion was applied with a supporting level of strength [11]. Loss of function variants were evaluated following the PVS1 (null variant) decision tree published by Abou Tayoun [14] using the AutoPVS1 online bioinformatics software [24] (http://autopvs1.genetics.bgi.com/). Computational data was assessed using REVEL [25] and SpliceAI (https://spliceailookup.broadinstitute.org). An exhaustive review of the literature was performed to obtain all available data from each variant regarding affected cases [23], functional [15, 26, 27], and segregation data [15, 16], or *hot spots* [15, 28, 29] and case-enriched regions [23, 30]. Our own patient’s database was also reviewed for affected cases carrying the evaluated variants.

The proportion of reclassified variants between the different cohorts was compared using the Chi-square test. When statistically significant differences were detected, a contrast of multiple comparisons was subsequently applied with the R software pairwise_prop_test function to determine which groups presented the differences. P values were regarded as statistically significant at p< 0,05.

Weighted Cohen’s kappa coefficient was used to measure the agreement between original classifications and the classifications after the reinterpretation performed in this study. The kappa values used to define limits were: no agreement (0 or less), poor (0.01–0.20), slight (0.21–0.40), fair (0.41–0.60), good (0.61–0.80), very good (0.81–0.92) or excellent (0.93–1.00) agreement [31].

## Results

### Variant reinterpretation in the inherited cardiovascular cohort

We reinterpreted 1425 genetic variants identified in 618 index cases genetically evaluated for cardiomyopathies, cardiac channelopathies, or SCD from 2012 to 2021. Of the 1425 genetic variants, 1131 were unique while 129 genetic variants were reported in multiple genetic analyses: 69, 14 and 1 were reported with the same classification in two, three or six genetic analyses, respectively; while 35, 7, 2 and 1 were reported with at least two different classifications in two, three, four and nine genetic reports, respectively.

**Fig 1:**
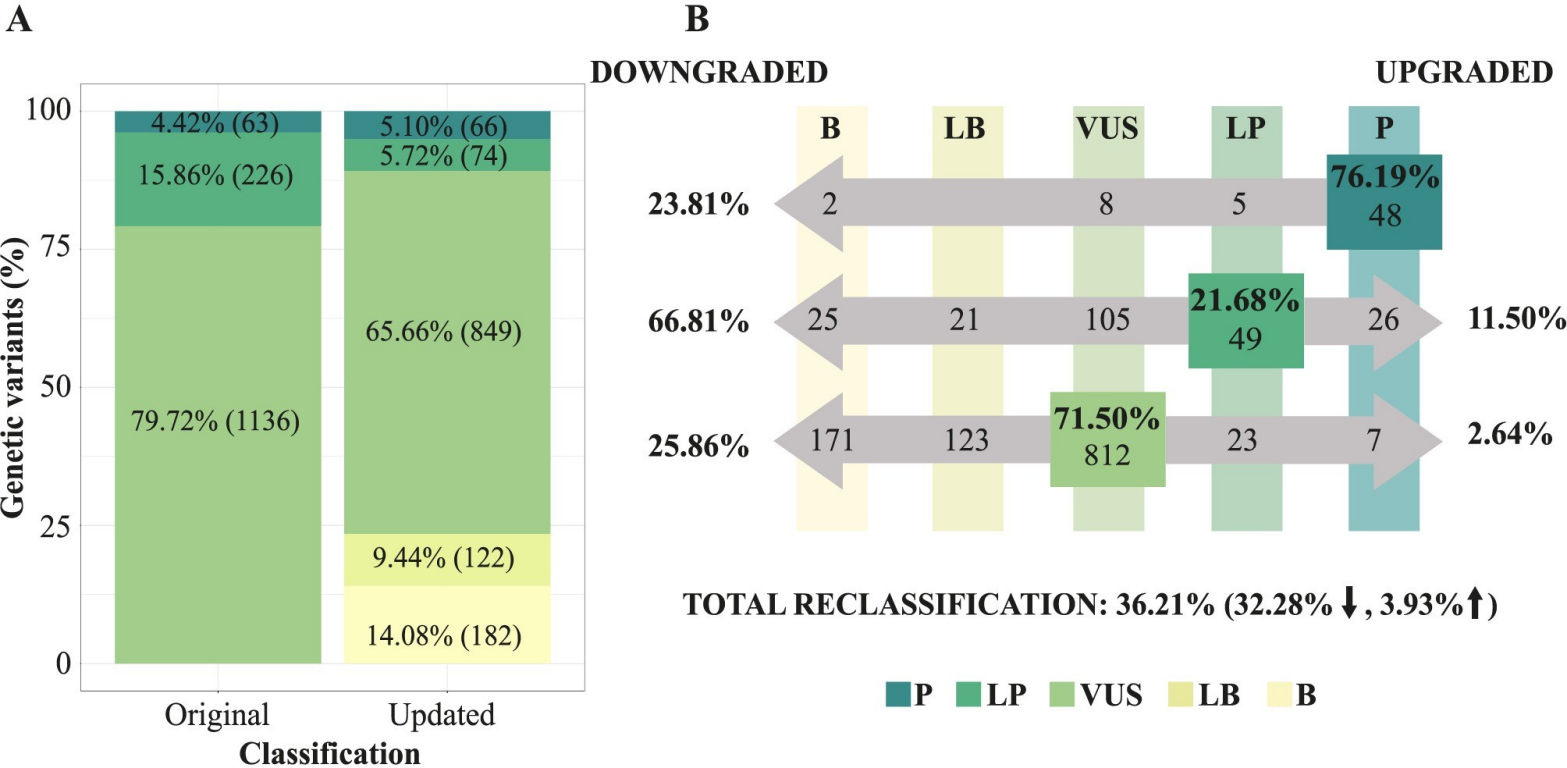
(A) Percentage and number of genetic variants for each of the classifications reported in the original report and after the reinterpretation. (B) Reclassification rate according to the original classification. The rows represent the variants that maintained or modified their classification after the reinterpretation for each original classification. On the left side, it is indicated the percentage of variants that reduced their classification for each original classification, and on the right side, the percentage that increased it. The columns show the number of variants for each classification after reinterpretation. P: Pathogenic; LP: Likely pathogenic; VUS: Variant of uncertain significance; LB: Likely benign; B: Benign.

Most genetic variants were missense (83.79%), followed by intronic variants (7.58%), loss of function variants (5.82%), in-frame indels (1.68%), copy number variants (0.63%), regulatory variants (0.28%) and synonymous variants (0.21%). Regarding the initially reported classification, 63 variants were classified as P (4.42%), 226 as LP (15.86%), and 1136 as VUS (79.72%) (Fig 1A). After reinterpretation, 81 variants were classified as P (5.68%), 77 as LP (5.40%), 925 as VUS (64.91%), 144 as LB (10.11%) and 198 as B (13.89%).

Globally, the reclassification rate was 36.21%, corresponding to 32.28% of reclassifications to classifications with a lower impact and 3.93% to classifications with a higher impact. Agreement analysis between initially reported and updated classifications using Cohen’s kappa coefficient showed a slight agreement (k = 0.2649).

As to variants initially reported as P, 76.19% of them maintained the classification after the reinterpretation (48 variants), while the remaining 23.81% were downgraded to LP (5 variants), VUS (8 variants) or B (2 variants) (Fig 1B). The 66.81% of the variants initially classified as LP were downgraded to VUS (46.46%, 105 variants) or LB/B (20.35%, 46 variants), while 21.68% maintained the LP classification (49 variants), and 11.50% were reclassified to P (26 variants). All P or LP variants that were reclassified to LB or B were initially classified before the publication of the ACMG guidelines. Regarding the 1136 variants initially reported as VUS, the majority retain the classification (71.50%, 812 variants), 25.86% were downgraded to LB/B (294 variants), and 2.64% acquired a LP/P classification (30 variants).

Considering those reclassifications that resulted in clinically relevant changes (P/LP to VUS or LB/B and VUS to P/LP or LB/B), the reclassification rate was 34.04%. Concretely, 31.93%(455/1425) and 2.11%(30/1425) of the genetic variants respectively lost or gained clinical relevance after reinterpretation, while 65.96%of them maintain their clinical relevance. Specifically, for those genetic variants originally reported as clinically actionable due to its classification as P or LP, 44.29% of them preserved this category, while 39.10% were downgraded to an uncertain clinical relevance (VUS) and the remaining 16.61% were downgraded to variants without clinical relevance (LB/B).

### Evidence that triggered reclassification of genetic variants

Table 1 shows the frequency with which each ACMG criterion was applied in variants that were reclassified during reinterpretation, providing potential evidence for the reclassification. Evidence was categorized as in the ACMG guidelines except for population and affected cases data and computation and loss-of-function (LOF) variants that each were split in two different categories, and segregation and *de novo* data that were considered together.

**Table 1:** Evidence applied on genetic variants reclassified during variant reinterpretation.

Type of evidence	% of genetic variants (n)	Specific criterium, % of variants (n)	
**Variants reclassified to higher impact classifications (VUS/LP to P and VUS to LP); n = 56**			
Population	100% (56)	PM2, 100% (56)	
Cases	60.71% (34)	PS4, 60.71% (34)	
Computational	42.86% (24)	PP3, 25% (14)	
		PM5, 14.29% (8)	
		PS1, 1.79% (1)	
		BP4, 1.79% (1)	
LOF	42.86% (24)	PVS1, 42.86% (24)	
Functional	55.36% (31)	PM1, 37.50% (21)	(PM1+PS3), 5.36% (3)
		PS3, 8.93% (5)	
		PP2, 3.57% (2)	
Segregation/*de novo*	42.86% (24)	PP1, 35.71% (20)	
		PS2, 7.14% (4)	
Other	10.71% (6)	PP4, 10.71% (6)	
**Variants reclassified to lower impact classifications (P to LP and P/LP to VUS); n = 118**			
Population	85.59% (101)	PM2, 71.19% (84)	
		BS1, 14.41% (17)	
Cases	13.56% (16)	PS4, 13.56% (16)	
Computational	57.63% (68)	PP3, 20.34% (24)	
		PM4, 4.24% (5)	
		PM5, 1.69% (2)	
		BP4, 10.17 (12)	(BP4+BP1), 10.17 (12)
		BP1, 11.02% (13)	
LOF	7.63% (9)	PVS1, 7.63% (9)	
Functional	12.71% (15)	PM1, 11.02% (13)	
		PP2, 1.69% (2)	
Segregation	4.24% (5)	PP1, 4.24% (5)	
**Variants reclassified to LB; n = 144**			
Population	100% (144)	BS1, 100% (144)	
Computational	97.92% (141)	BP4, 51.39% (74)	(BP4+BP1), 31.25% (45)
		BP1, 15.28% (22)	
Functional	1.39% (2)	BS3, 1.39% (2)	
Segregation	0.69 (1)	BS4, 0.69 (1)	
**Variants reclassified to B; n = 198**			
Population	100% (198)	BA1, 100% (198)	

**Fig 2:**
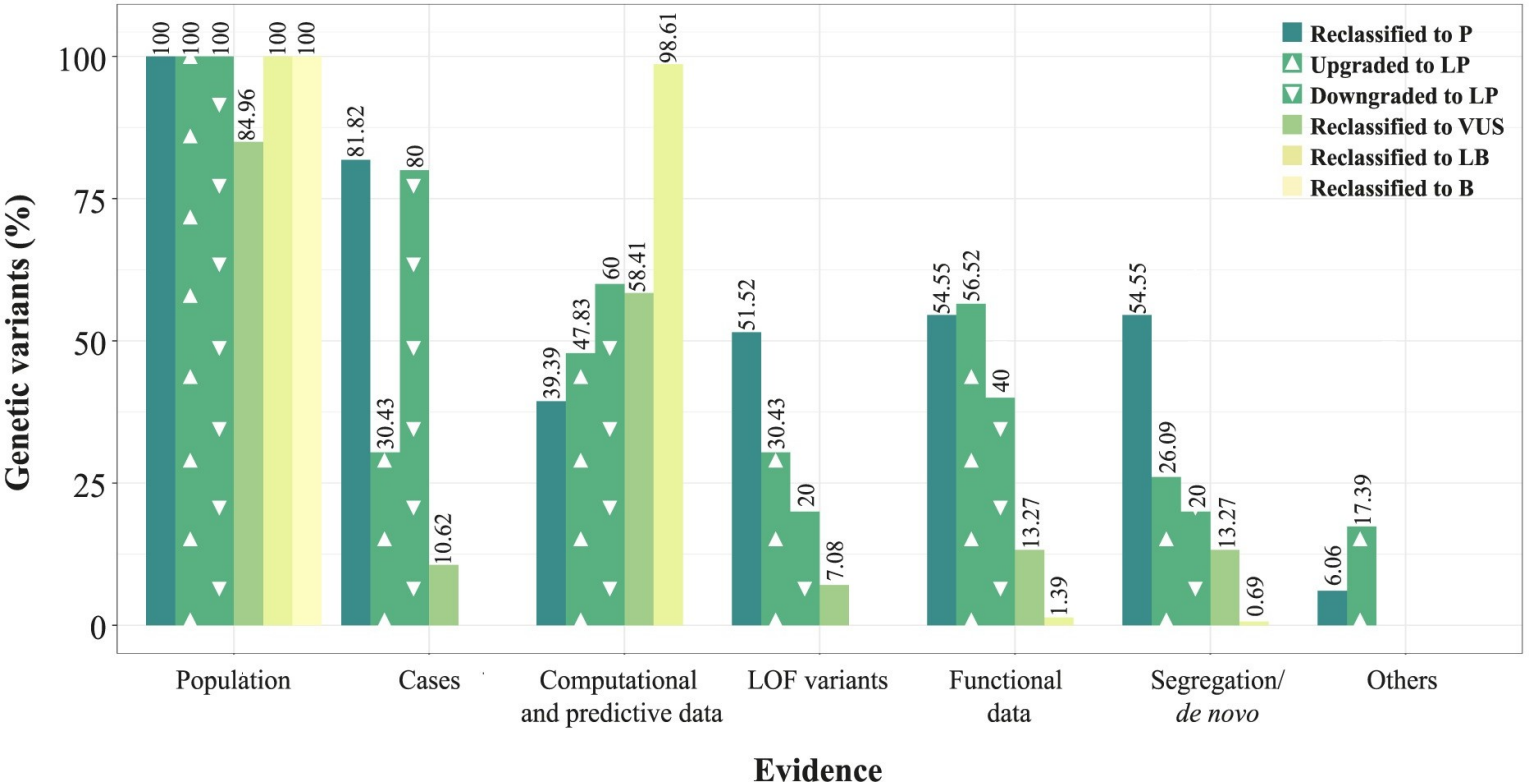
Proportion of genetic variants in which each type of evidence was applied during reinterpretation. Variants are grouped in: variants reclassified to P, VUS, LB or B, variants upgraded to LP and variants downgraded to LP.

All genetic variants that were reclassified to higher impact classifications (VUS/LP to P and VUS to LP) were absent in general population databases or had an extremely low allelic frequency, allowing the fulfilling of the PM2 criterium. Evidence of affected cases was the second most applied criterion in 60.71%of variants reclassified to higher impact classifications during reinterpretation, followed by 55.36% with functional data, and 42.86% who fulfilled segregation/*de novo* criteria, 42.86% were LOF variants and also in 42.86% of the variants one of the computational and predictive data evidence were applied such as *in silico* predictions (PP3) or evidence of different changes at the same amino acid residue (PM5) or the same amino acid change (PS1) being described before as P. Finally, in 10.71%of the variants the supporting evidence for the specificity of the patient’s phenotype with the gene was applied (PP4). As shown in Fig 2, differences were observed between the proportion of variants reclassified to P and the proportion of variants upgraded to LP to which evidence from affected cases (81.82% vs 30.43%), LOF variants (51.52% vs 30.43%), and segregation data (54.55% vs 26.09%) was applied, being higher in the variants reclassified to P.

As for variants that were reclassified to lower impact classifications after reinterpretation (P to LP and P/LP to VUS), 71.19% also fulfilled the PM2 criterium for absence or extremely low allelic frequency in general population databases while in 14.41% the allelic frequency was higher than expected for the disease (BS1). In 57.63% of the variants a computational or predictive criterium was applied however, this proportion consisted in 31.36% of variants in which the applied evidence supported the benignity of the variant and the remaining 26.27% supported the pathogenicity of the variant. Other evidence such as affected cases, functional and segregation data and LOF variants were applied in less than 14% of the variants reclassified to lower impact classifications. However, these were applied in a much higher proportion in variants downgraded to LP during reinterpretation than in those downgraded to VUS: 80% vs 10.62% of variants fulfilled evidence from affected cases, 20% vs 7.08% of LOF variants, 40% vs 13.27% of functional data and 40% vs 2.70% of segregation data, respectively. In fact, the proportion of variants downgraded to LP which fulfilled evidence about affected cases, LOF variants, and segregation and functional data was more similar to those variants reclassified to P than those downgraded to VUS (Fig 2).

Variants reclassified to LB and B were also variants that were reclassified to lower impact classifications, however, were evaluated separately due to the distinctive characteristics of the evidence applied in their reinterpretation. In all the variants reclassified to LB the BS1 criteria was applied as their allelic frequency in general population databases was higher than expected for the disease. In 97.92% of variants reclassified to LB a benign computational and predictive evidence was applied together with BS1, while in the remaining, lack of segregation or no functional effect of the variant helped achieve the LB classification.

In all variants reclassified as B, evidence of an allelic frequency too high for considering them disease associated (BA1) was applied as a stand-alone criterium.

### Variant reinterpretation in the cardiomyopathy vs cardiac channelopathy cohorts

As the 1425 genetic variants reinterpreted included variants identified in individuals evaluated for cardiomyopathies, cardiac channelopathies, and SCD, it was suggested that reclassification rates could vary according to the specific patient’s phenotype associated with the genetic variants being reinterpreted. Therefore, variants were categorized into: those identified in patients with cardiomyopathies (cardiomyopathy cohort) and those identified in patients with cardiac channelopathies (channelopathy cohort). From these selections, we excluded those variants identified in SCD cases in which a definitive diagnosis of cardiomyopathy or cardiac channelopathy could not be established.

**Fig 3:**
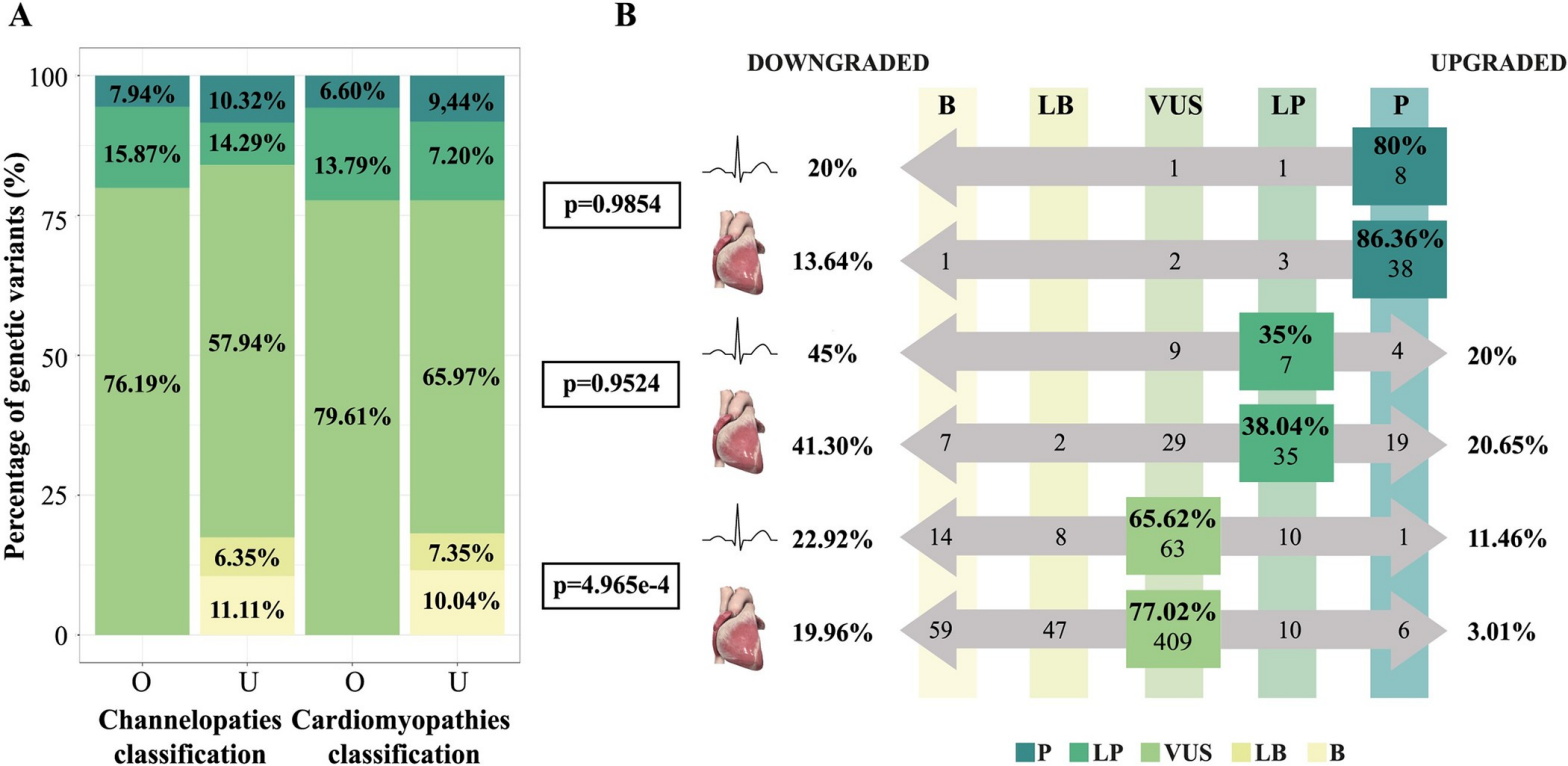
(A) Percentage of genetic variants for each of the classifications reported in the original report and after the reinterpretation in the cohort of cardiac channelopathies and cardiomyopathies. (B) Reclassification rate according to the original classification of variants included in the cardiac channelopathies’ and cardiomyopathies’ cohorts. Cardiac channelopathies’ cohort is represented with an ECG and cardiomyopathies’ cohort is represented with a heart image. The rows represent the variants that maintained or modified their classification after the reinterpretation for each original classification. On the left side, it is indicated the percentage of variants that reduced their classification for each original classification, and on the right side, the percentage that increased it. The columns show the number of variants for each classification after reinterpretation. O: Original; U: Updated; P: Pathogenic; LP: Likely pathogenic; VUS: Variant of uncertain significance; LB: Likely benign; B: Benign.

The cardiomyopathy and channelopathy cohorts included 667 and 126 genetic variants, respectively. Variant proportions for each classification (original and updated) are shown in Fig 3A. Globally, the cardiomyopathy cohort reclassification rate was27.74%, with 22.49% of variants being reclassified to a lower impact classification and 5.25% to higher impact classification. The channelopathy cohort showed a reclassification rate of 38.10%, corresponding to a 26.19% downgrade of the original classification and a 11.90% upgrade. Comparison analyses demonstrated significant differences in reclassification rates between both studied cohorts (p = 7.87e-3), showing a significantly higher proportion of variants that upgraded their classification in the channelopathy cohort (p = 8.8e-4; 11.90% vs 5.25%;while the proportion of variants that maintain the original classification after reinterpretation is higher in the cardiomyopathy cohort (p = 0.0254; 72.26% vs 61.90%). No significant differences were observed in the proportion of genetic variants that downgraded their classification between the cardiomyopathy and channelopathy cohorts (p = 0.430; 22.49% vs 26.19%).

The reclassification rate was evaluated considering the initial classification for each cohort to determine if the statistically significant differences affected all classifications (Fig 3B). As to variants initially reported as P in the channelopathy and cardiomyopathy cohorts, 80% vs 86.36% maintained the P classification, respectively, while 20% vs 13.64% of P variants downgraded their classification in one or more levels. Therefore, no significant differences were observed in the reclassification of variants initially reported as P between the channelopathy and the cardiomyopathy cohorts (p = 0.9854).

No differences were observed in the reclassification of variants initially reported as LP between cohorts (p = 0.9524). In the channelopathy cohort, 45% of the variants were reclassified to a classification with lower impact, 35% remained classified as LP, and 20% of the variants acquired a P classification. In the cohort of cardiomyopathies, 41.30% were reclassified to a classification with lower impact, 38.04% maintained the LP classification, and 20.65% of the variants were reclassified to P.

Finally, reclassification rates of variants initially reported as VUS showed statistically significant differences between the cardiomyopathies cohort and the channelopathy cohort (p = 4.964e-47). Specifically, 22.92% of variants initially reported as VUS in the channelopathy cohort and 19.96% in the cardiomyopathy cohort were downgraded to LB/B (p = 0.601), while a significantly higher proportion of variants from the cardiomyopathy cohort preserved the VUS classification (p = 5.05e^-4^; 77.02% vs 65.62%) and the proportion of variants reclassified to P/LP was significantly higher in the channelopathy cohort (p = 0.0242; 11.46% vs 3.01%). Further evaluation of VUS reclassified to P/LP variants showed statistically significant differences between VUS reclassified to LP in the channelopathy and cardiomyopathy cohorts (p = 4.84e^-5^; 10.42% vs 1.88%), while no differences were observed in those reclassified to P (p = 1; 1.04% vs 1.13%).

### Variant reinterpretation according to the original classification’s year

**Fig 4:**
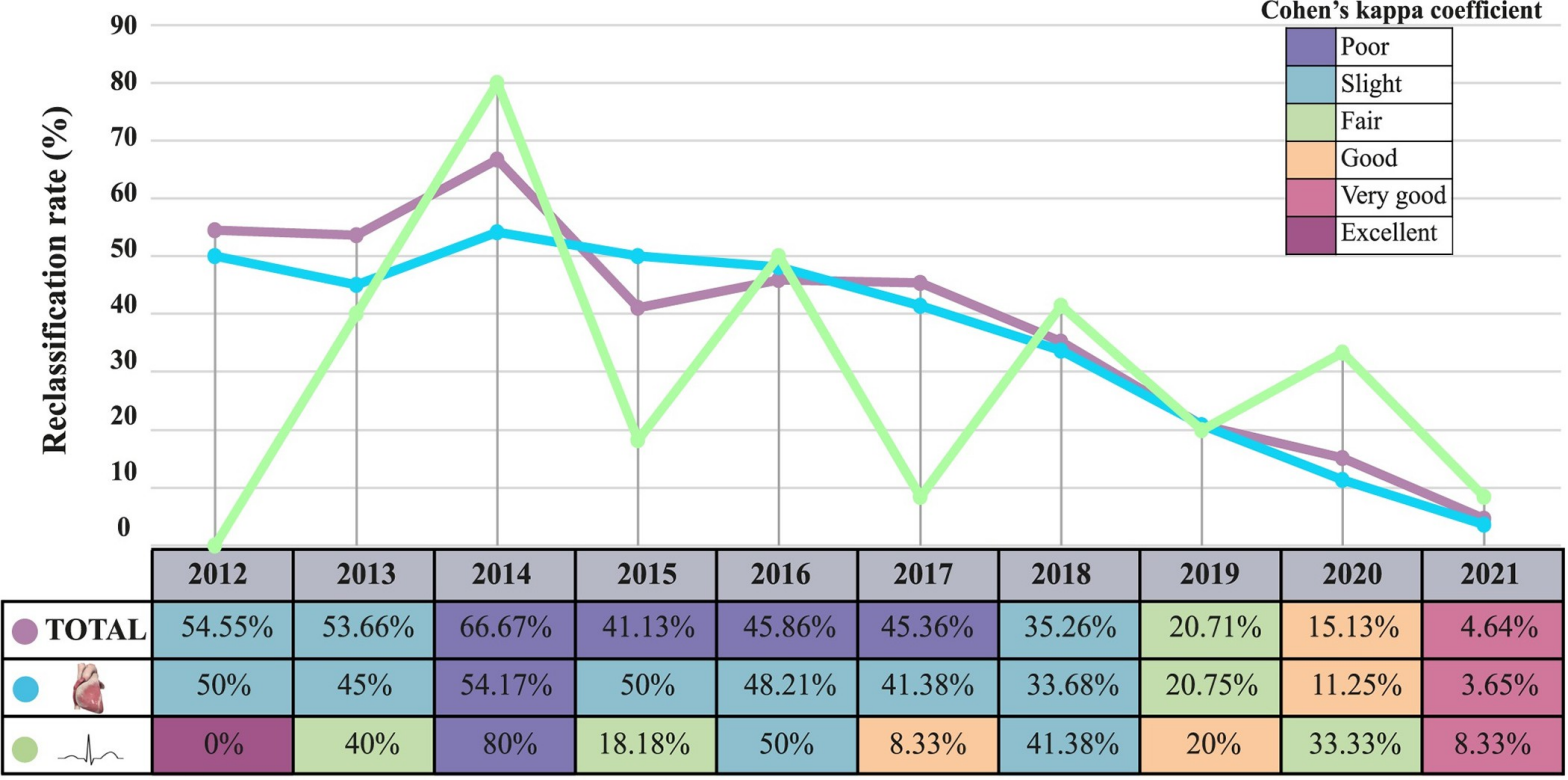
Reclassification rates for the 1425 variants (total cohort of inherited cardiovascular diseases) and the cohorts of cardiomyopathies and cardiac channelopathies and agreement according to the Cohen’s coefficient between the original and updated classifications depending on the original year of classification for each cohort.

Agreement analysis between initially reported and updated classifications of the 1425 genetic variants evaluated in this study showed a slight agreement (k = 0.2649). However, it was suggested that the agreement analysis between initial classification and classification after reinterpretation would be better the shorter the time since the initial reporting. Therefore, the impact of reinterpretation was assessed based on the time since the genetic variant was initially reported using the 1425 genetic variants (total cohort) and the variants from the cardiomyopathy and channelopathy cohorts. Reclassification rates and results of the agreement analysis by year of the initial reporting for the three cohorts are described in Fig 4.

As for the 1425 variants included in the study, reclassification rates of variants reported until 2017 (5 or more years before reinterpretation) were higher than 40%, while in 2018 it started to decrease, starting with a reclassification rate of 35.26%in variants reported in 2018 and reaching 4.64% in the variants reported in 2021 (one year before reinterpretation). The agreement analysis showed "poor" and "slight" results between the original classification and the classification after reinterpretation in variants initially reported more than 4 years before reinterpretation (between 2012 and 2018). In those variants reported in 2019 (3 years before reinterpretation), the agreement between classifications increased to "fair" (k = 0.4249). It continued improving to "good" in variants reported in 2020 (k = 0.6520) and to "very good" in variants reported in 2021 (k = 0.8938).

Results from the cardiomyopathy cohort were similar to those of the total subset of variants. Reclassification rates of variants reported yearly from 2012 to 2017 were above 40%. However, in those variants reported less than 4 years before reinterpretation, yearly reclassification rates started to decrease, reaching a reclassification rate of 3.65% in those reported in 2021, one year before reclassification. As for the agreement analysis between original and updated classifications, it was "poor" or "slight" until 2019, when a "fair" agreement was observed (k = 0.4788). Variants initially reported in 2020 reached a “good” agreement (k = 0.7793) and a "very good" agreement was reached in variants reported in 2021 (k = 0.9221).

Variants from the cardiac channelopathy cohort showed more fluctuant reclassification rates, alternating between years with reclassification rates above 50% and years with reclassification rates below 20%. Agreement analysis also showed alternating results in those years before 2019 with "poor", "slight", "fair" and even “good” results, but only variants initially reported in 2021 reached a “very good” agreement (k = 0.8938).

### Variant reinterpretation in genes with definitive clinical validity

Reclassification rates in different cardiomyopathies (HCM, DCM and ACM) and cardiac channelopathies (BrS, LQTS, and CPVT) were evaluated focusing on the clinical validity of the genes where genetic variants were identified.

#### Cardiomyopathy subcohorts

In the HCM subcohort, genetic testing was performed in 224 index cases identifying a total of 420 genetic variants in 185 of them: 143 in genes with a definitive clinical validity to HCM (*ACTC1*, *MYBPC3*, *MYH7*, *MYL2*, *MYL3*, *TNNI3*, *TNNT2* and *TPM1*) and in syndromic genes in which left ventricular hypertrophy is observed either isolated or together with other syndromic features (*ABCC9*, *BAG3*, *CRYAB*, *DES*, *FLNC*, *GAA*, *GLA*, *LAMP2*, *LMNA*, *PRKAG2* and *TTR*), 20 in genes with moderate clinical validity (*ACTN2*, *CSRP3*, *JPH2* and *TNNC1*), 145 in genes with limited clinical validity and 112 in genes with no association to HCM.

In the DCM subcohort, 93 index cases were genetically analyzed, identifying a total of 185 genetic variants in 85 of them: 99 in genes with a definitive clinical validity to DCM (*DES*, *DMD*, *FKTN*, *FLNC*, *LAMP2*, *LMNA*, *MYH7*, *RBM20*, *SCN5A*, *TNNT2* and *TTN*), 6 in a gene with strong clinical validity (*DSP*), 14 in genes with a moderate clinical validity (*ACTC1*, *ACTN2*, *JPH2*, *NEXN*, *TNNI3* and *VCL*), 21 in genes with limited clinical validity and 45 in genes with a disputed/refuted association or no association to DCM.

Finally, in the ACM subcohort, 40 index cases were genetically analyzed, identifying a total of 82 genetic variants in 36 of them: 27 in genes with a definitive clinical validity to ACM (*DSG2*, *DSP*, *FLNC*, *JUP*, *PKP2* and *TMEM43*), 1 in a gene with moderate clinical validity (*ACTN2*), 19 in genes with limited clinical validity and 35 in genes with disputed/refuted association or no association to ACM.

**Fig 5:**
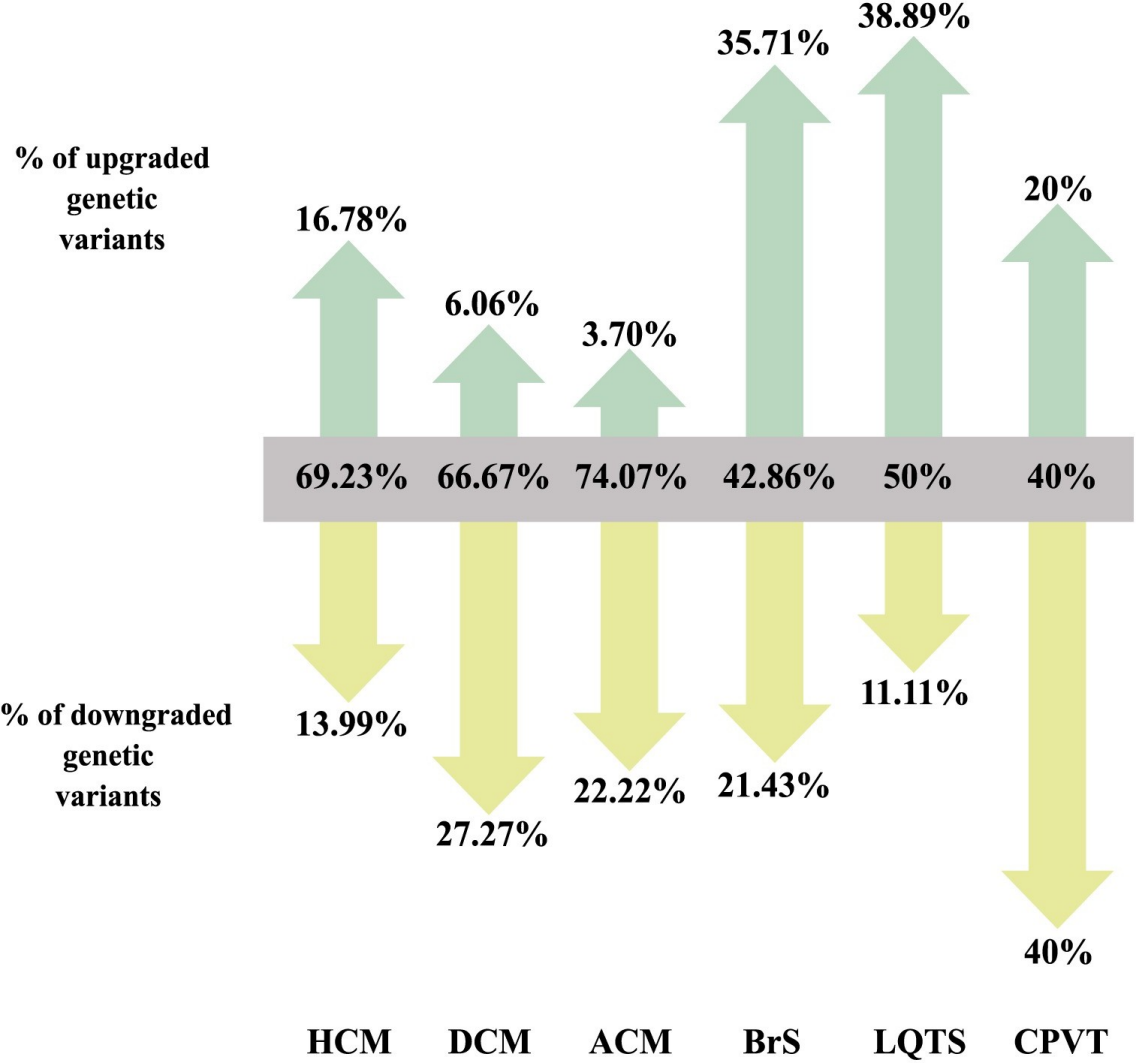
Percentage of genetic variants that downgraded, maintained, or upgraded the original classification after reinterpretation for the cardiomyopathies (HCM, DCM and ACM) and cardiac channelopathies subcohorts (BrS, LQTS and CPVT).

Reclassification rates for those variants identified in genes with a definitive clinical validity were 30.77% in the HCM subcohort, 33.33% in the DCM subcohort and 25.93% in the ACM subcohort (Fig 5). In the HCM subcohort, a superior reclassification rate to higher impact classifications (16.78%) was observed compared to the reclassification rate to lower impact classifications (13.99%). In contrast, in the DCM and ACM subcohorts, the proportion of variants that downgraded their classification due to reinterpretation was significantly higher than the proportion of variants that upgraded the classification (27.27% vs 6.06% and 22.22% vs 3.70%, respectively). Lack of evidence supporting the original classification was one of the main reasons that triggered reclassification to lower impact classification, followed by the fulfilling of benign criteria due to high allelic frequency in general population databases (BA1 for all variants reclassified to B and BS1 for all variants reclassified to LB). On the other hand, evidence that led to reclassifications to higher impact classifications were primarily segregation and the availability of other affected cases carrying the same variant.

All genetic variants identified in genes with clinical validities other than definitive were reclassified to VUS, LB, or B except for five variants identified in genes with no association to the patient’s phenotype: two *PKP2* variants identified in patients with HCM and DCM, which were considered P in an ACM context; a *KCNQ1* variant identified in a patient with HCM, which vas considered P in a LQTS context; one variant in *FKTN* identified in a patient with HCM and considered P in a context of autosomal recessive muscular dystrophy and a *SCN5A* variant identified in a patient with HCM and considered LP in a BrS context.

Additionally, reclassification rates were compared between LOF and non-LOF genetic variants in genes with a definitive clinical validity for the HCM, DCM and ACM subcohorts. In the HCM subcohort most of the LOF variants were identified in the *MYBPC3* gene (22/24 LOF variants). Eight out of the 24 LOF variants were reclassified to P (33.33%) while the remaining retained the original classification. In the DCM subcohort also 8 out of the 24 LOF variants were reclassified after reinterpretation, resulting in a 33.33% reclassification rate of which 25% were variants reclassified to higher impact classifications and 8.33% were variants reclassified to lower impact classifications. LOF variants in the DCM subcohort were identified mainly in the *TTN* gene (19/24 LOF variants). Finally, the 5 LOF variants in *PKP2*, *DSP* and *FLNC* identified in the ACM subcohort maintained their original P or LP classification.

As for non-LOF, the reclassification rate was 30.25%; 33.33% and 31.82% in the HCM, DCM and ACM subcohorts, respectively. In the DCM subcohort, all reclassified non-LOF variants acquired a lower impact classification, while in the HCM 16.81% of the non-LOF variants were reclassified to a classification with higher impact and 13.45% of the non-LOF variants were reclassified to a classification with lower impact, and in the ACM subcohort. 27.27% of the non-LOF variants were downgraded and 4.55% were upgraded.

#### Cardiac channelopathy subcohorts

In the BrS subcohort, genetic testing was performed in 62 index cases identifying a total of 70 genetic variants in 43 of them: 14 in a gene with a definitive clinical validity to BrS (*SCN5A*), 28 in genes with disputed clinical validity and 28 in genes with no association to BrS.

In the LQTS subcohort, 26 index cases were genetically analyzed, identifying a total of 62 genetic variants in 24 of them: 18 in genes with a definitive clinical validity to LQTS (*KCNH2*, *KCNQ1*, *SCN5A*, and *CALM1*), 1 in a gene with a moderate clinical validity (*CACNA1C*), 1 in a gene with limited clinical validity (*KCNE1*), 4 in a gene with disputed clinical validity (*ANK2*) and 17 in genes with no association to LQTS.

In the CPVT subcohort, 5 index cases were genetically analyzed, identifying 14 genetic variants: 4 in a gene with a definitive clinical validity to CPVT (*RYR2*) and 9 in genes with no association to CPVT.

Evaluation of reclassification rates for those variants identified in genes with a definitive clinical validity showed a reclassification rate of 57.14% in the BrS subcohort, 50% in the LQTS subcohort and 60% in the CPVT subcohort. In the BrS and LQTS subcohorts, the rate of reclassification to higher impact classifications was superior compared to that of reclassification to lower impact classifications (35.71% vs 21.43% and 38.89% vs 11.11%, respectively). In the CPVT cohort, 40% of the variants were downgraded, while 20% were upgraded (Fig 5). The main reasons that triggered reclassifications to classifications with lower impact were primarily the lack of evidence to support the original classification and the high allelic frequency in general population databases. In contrast, among the evidence that led to an upgrade in the classification, we find evidence of segregation or affected cases, the localization of the variant in a hot spot, or the availability of functional studies.

As for genetic variants identified in genes with clinical validities other than definitive, all were reclassified to VUS, LB, or B except for two variants identified in genes with no association to the patient’s phenotype: a *KCNQ1* variant identified in a patient with BrS, which vas considered LP in a LQTS context; and a *PKP2* variant identified in a patient with LQTS, which was considered P in an ACM context.

## Discussion

A misclassification of a genetic variant can lead to an inappropriate genetic diagnosis and the adoption of unnecessary or inadequate therapeutic measures in both the patient and their relatives. Therefore, one of the main challenges of genetic analysis is the interpretation of the identified variants. As classifications are obtained by combining all the variant evidence existing at the time of its evaluation, they are subject to changes over time due to potential new available evidence, indicating the need for periodic reinterpretation.

Currently, the debate is focused on the frequency with which reinterpretation of reported variants should be carried out, who should initiate the reinterpretation process, how the patient should be recontacted, and the role of the patient’s consent in the process of reinterpretation, as well as the financial implications and the extra workload it entails. However, the potential benefit for the patients and their relatives suggests the importance of reaching a consensus on these aspects of reinterpreting genetic variants. In this study, genetic variants identified in a cohort of familial heart diseases for ten years have been reinterpreted to determine the timeframe for reinterpretation and the reclassification impact on different inherited cardiovascular disease cohorts.

The reinterpretation of 1425 genetic variants identified in patients evaluated for cardiomyopathies, cardiac channelopathies, or SCD between 2012 and 2021 showed a reclassification rate of 36.21%. Similar values have been reported in other studies which evaluated reinterpretation of genetic variants identified in survivors of cardiac arrest (37.98%) [32] or in patients with pediatric epilepsy (36.30%) [19], while is higher than the reclassification rate described in dilated cardiomyopathy (29.82%) [33] or hereditary cancer cohorts (3.60%-12.40%) [34–38]. We also observed reclassification rate divergences between the different cohorts evaluated in this study: cardiomyopathies (27.74%) and cardiac channelopathies (38.10%). Furthermore, we demonstrated higher reclassification rates (50%-60%) in those cohorts in which reinterpretation was restricted to variants identified in genes showing a definitive association with the specific cardiac channelopathy presenting on the patient (BrS, LQTS and CPVT). However, when evaluating those variants identified in genes with a definitive clinical validity to the different cardiomyopathies entities (HCM, DCM and ACM), reclassification rates were even lower than when evaluated all variants from the inherited cardiovascular cohort (25–30%). Therefore, the difference between cardiomyopathies and channelopathies remains even when we evaluate only the variants identified in genes with a definite clinical association. One of the reasons associated with these differences could be that the diagnosis of cardiomyopathies is easier to achieve, and they often present with family history, which increase the frequency with which segregation data is available. At the same time, cardiomyopathies have a higher prevalence, thus, more information about cases and families is available in the scientific literature and databases. Additionally, specific ACMG guidelines for cardiomyopathies were already published in 2018 [15] while for cardiac channelopathies there are not specific guidelines yet except for concrete specifications about hot spots of the main genes associated to BrS and LQTS [23].

Contrarily, both in cardiomyopathies and cardiac channelopathies, genetic variants in genes with no association to the disease generally maintain the VUS classification or are reclassified to LB/B. Thus, it can be concluded that apart from the availability of new specific data on the genetic variant, such as data from cases and segregation, accessibility to specific evidence regarding the disease or the associated genes is crucial.

Regarding the main evidence used for reclassification of variants, it was observed that the availability of information about other affected cases was one of the most applied criteria in variants with clinical relevance, as well as loss-of-function evidence and segregation and functional data. This corresponds with the ACMG criteria that present different levels of strength depending on the available evidence, so their application allows for higher impact classifications. Concerning the observed high rate of reclassifications to classifications with lower impact, the implementation of the ACMG guidelines caused the reclassification of many variants whose pathogenicity had been overestimated and that currently do not meet sufficient criteria to maintain the original classification. Additionally, the publication of databases such as gnomAD and especially the popmax feature permitted the reassessment of variants with much more robust data on allelic frequency, allowing to rule out many variants that are considered benign due to their high allelic frequency in these databases.

Reclassification rate is intimately associated with the studied cohort, the clinical characteristics of patients, the clinical validity of genes, and the number of variants included in the reinterpretation. Moreover, reclassification rates will vary depending on whether variants were initially classified following the ACMG guidelines. It may also depend on whether reinterpretation was performed before or after the publication and updates of population databases such as ExAC or gnomAD. Hence, multiple factors can influence different reclassification rates. In this study, we evaluated the reinterpretation rate according to the initial reporting year of the genetic variants to establish the maximum periodicity with which the genetic variants should be reinterpreted. Different authors have stated that it should be executed every 2 [19] or 5 years [17, 18]. However, the studies conducted by our group were based on the reinterpretation of genetic variants 10 and 5 years after they were initially reported in 2010 [17] or 2016 [18], respectively, but did not evaluate the impact of the reinterpretation in different years to be able to determine the most appropriate frequency for the reinterpretation; while SoRelle [19] reinterpreted the genetic variants reported 2 and 5 years before, showing a lower rate of reclassification in those reported during the last 2 years [19]. Chisholm [39] proposed a more detailed approach for reinterpretation: 1) upon health professional request, 2) genetic variants identified in an unrelated proband and it has been >12 months since the last evaluation of this variant, and 3) an extension of a genetic study is requested, and it has been > 12 months since variants in the first report were classified [39]. Our study is the first to evaluate the reinterpretation impact from a subset of near 1500 genetic variants according to the reported year, from 2012 to 2021, to determine the most appropriate frequency for reinterpretation.

Reinterpretation results indicated reclassification rates between 35–70% for those genetic variants reported before 2019, variants reported in 2019 and 2020 showed slightly lower values, and a reclassification rate of less than 10% was achieved in variants reported with a maximum of 1 year before the reinterpretation. Agreement analysis between initially reported and updated classifications using Cohen’s kappa coefficient was "good" in those genetic variants reported in 2020 and 2021, while in those variants reported the previous years oscillated between "poor", "slight", "fair". The evaluation of reinterpretation depending on the initially reported year was also performed for the cardiac channelopathies and cardiomyopathies subcohorts. The cardiomyopathies subcohort showed similar results, while in the cardiac channelopathies subcohort, both reclassification rates and the agreement analysis results fluctuated from values between 10–80% and “poor” and “good” agreements between original and updated classifications. Those divergent results could be explained due to specifications published in 2021 regarding the regions enriched with pathogenic variants in the main clinically relevant genes associated to cardiac channelopathies [23], which contributed to higher reclassification rates in this subcohort for those years with high percentage of variants in such genes. Global results showed a "good" agreement only in those reinterpretations performed in genetic variants reported with a maximum of 2 years before reinterpretation, indicating this could be the most appropriate frequency for the reinterpretation of genetic variants identified in individuals evaluated for inherited cardiovascular diseases. However, in some cases, a more often reinterpretation could be beneficial due to the publication of new specifications for the appropriate use of evidence or availability of segregation data, among others, which could cause a variant to modify its classification even before two years.

It should be noted that, although reclassifications to lower impact classification of variants originally classified as P have been described in this study, if a recent classification following the ACMG guidelines has been performed, it would not be necessary to periodically reinterpret P variants since they are not expected to vary the classification. In the absence of major changes to variant interpretation such as the implementation of the ACMG guidelines or the emergence of gnomAD, reclassification rates for VUS and LP recently classified following the ACMG guidelines should be lower. Reclassification would primarily occur when new evidence becomes available, such as data from segregation studies or potential future ClinGen gene-disease evaluations.

Variant reclassification may have important implications in clinical care. Genetic testing provides several benefits such as diagnostic precision, therapeutic options, and information on prognosis. However, if genetic variants are misclassified, clinical care decisions could be incorrectly implemented either by overtreating or wrongly discharging patients. An honest discussion about the limitations of genetic testing has to be established with the families undergoing genetic testing so as to understand the implications of variant reclassification as new information becomes available or guidelines are updated. Reclassification of the genetic variants in diseases associated with sudden cardiac death may bring psychosocial issues that will need to be addressed with specialized genetic counseling.

Genetic counselors are responsible for providing, to the patient and family members, all the information regarding genetic testing and the implications of each possible result as well as the possibility of reinterpretation if new evidence becomes available. It has been described that a high percentage of genetic counselors working in specialized inherited cardiovascular disease units take responsibility for genetic variant classification, expanding their skills as specialist genetic counselors in cardiovascular disease and providing expertise to multidisciplinary teams [40]. In addition, the involvement of genetic counselors in the variant reinterpretation process allows genetic variants to be reassessed more frequently, granting the possibility of immediately incorporating potential changes into patient surveillance and family risk assessment. Therefore, we propose that genetic counselors take an active role in the reinterpretation process and, if necessary, request the variant to be reinterpreted by the diagnostic laboratory. It is also proposed that the diagnostic laboratory performs variant reinterpretations whenever there is a healthcare provider request. Additionally, every time a genetic report from a previously reported variant is issued, either from a different index case or from family members, new potentially available evidence should be reviewed to ensure the variant classification is updated. If variant reinterpretation results in a reclassification, the diagnostic laboratory should notify the requesting healthcare professional and genetic counsellors should be included in providing this information to the patient and family members.

In conclusion, accurate interpretation of genetic variants is essential to achieve the correct genetic diagnosis and provide the appropriate therapeutic measures to the patient and family members. Yearly reclassification rates of genetic variants identified from 2012 to 2021 in patients evaluated for cardiomyopathies, cardiac channelopathies, or SCD are above 25% in all the studied cohorts. However, in cardiac channelopathies it is even higher when only variants identified in genes with clinical validity are evaluated. Those high reclassification rates highlight the need to implement the reinterpretation of genetic variants periodically. This study estimates that variants should be reinterpreted two years after their last evaluation, with the possibility of a more frequent reinterpretation if considered appropriate.

## Supporting information

S1 Table List of genetic variants evaluated in the reinterpretation. (XLSX)
